# T-type Ca^2+^ channels and their relationship with pre-neoplastic and neoplastic lesions in the human breast

**DOI:** 10.1590/1414-431X2023e11879

**Published:** 2023-02-10

**Authors:** F. Aguiar, P. Rhana, E. Bloise, C.B. Nunes, A.L. Rodrigues, E. Ferreira

**Affiliations:** 1 Departamento de Patologia, Instituto de Ciências Biológicas, Universidade Federal de Minas Gerais, Belo Horizonte, MG, Brasil; 2 Programa de Imunologia e Biologia Tumoral, Instituto Nacional de Câncer, Rio de Janeiro, RJ, Brasil; 3 Department of Physiology and Membrane Biology, University of California Davis, Davis, CA, USA; 4 Departamento de Morfologia, Instituto de Ciências Biológicas, Universidade Federal de Minas Gerais, Belo Horizonte, MG, Brasil; 5 Departamento de Anatomia Patológica e Medicina Legal, Faculdade de Medicina, Universidade Federal de Minas Gerais, Belo Horizonte, MG, Brasil; 6 Departamento de Bioquímica e Imunologia, Instituto de Ciências Biológicas, Universidade Federal de Minas Gerais, Belo Horizonte, MG, Brasil

**Keywords:** Breast cancer, Ductal hyperplasia, Carcinoma *in situ*, T-type voltage-dependent Ca^2+^ channel, Neoplastic transformation

## Abstract

The expression of T-type voltage-dependent Ca^2+^ channels (Cav3) has been previously observed in breast cancer, but their expression and subcellular localization were not evaluated in pre-neoplastic lesions. Therefore, this work aimed to evaluate protein expression and subcellular localization of T-type channel isoforms in human breast tissue samples. Protein expressions of Ca_V_3.1, Ca_V_3.2, and Ca_V_3.3 were evaluated by immunohistochemistry in breast without alteration, in proliferative non-neoplastic lesions, and in neoplastic ductal epithelial lesions of the human breast. Ca_V_3.1, Ca_V_3.2, and Ca_V_3.3 nuclear expressions were decreased in advanced stages of neoplastic transformation, whereas Ca_V_3.1 and Ca_V_3.2 cytoplasmic expression increased. Also, the decrease in nuclear expression was correlated with an increase in cytoplasmic expression for Ca_V_3.1 isoform. The change in Ca_V_3 protein expression and subcellular localization are consistent with the neoplastic transformation stages of mammary epithelial cells, evident in early neoplastic lesions, such as ductal carcinomas *in situ*. These results suggest a possible involvement of Ca_V_3 in the carcinogenic processes and could be considered as a potential pharmacological target in new therapies for breast cancer treatment.

## Introduction

Among the various types of cancer, breast cancer has the highest incidence among women and accounts for the largest number of deaths in women, with 627,000 deaths in 2018 (1). Because breast cancer is a heterogeneous disease with distinct histological and biological features, there are a variety of clinical presentations and behaviors, as well as different responses to available therapies.

The most common breast cancer subtype is invasive ductal carcinoma (IDC) (2). Historically, neoplastic transformation to this subtype has been traced as a stepwise model, similar to Vogelstein's model for colon cancer. The first step is usually ductal hyperplasia that can transform into atypical hyperplasia, which in turn can be transformed into ductal carcinoma *in situ* (DCIS), and finally, into IDC. Although these lesions are considered possible non-obligate precursors of invasive ductal carcinoma (3,4), due to the lack of an efficient biomarker to identify cases prone to this evolution, women undergo treatment excessively (5,6).

It is well established that non-obligate precursor lesions have a high risk of developing into breast ductal carcinoma. Patients diagnosed with common hyperplasia are twice as likely to develop IDC, while those with atypical hyperplasia are four to five-fold more likely to have this transformation (7,8). Women diagnosed with DCIS that remain untreated have a ten-fold higher risk of developing IDC (6). In addition, these lesions have in common that the number of epithelial cells is increased due to changes in proliferation (9). Changes in this cellular process are known to be closely related to a greater probability of transformation, since lesions with high proliferation rates are more likely to become malignant compared to those with lower rates (10).

One of the regulatory mechanisms for cell proliferation is controlled by intracellular Ca^2+^ levels, a very important and ubiquitous intracellular second messenger related to several functions, such as proliferation, cell cycle, apoptosis, gene transcription, and migration (11). In cancer, Ca^2+^ signaling contributes to a favorable tumor microenvironment, with uncontrolled production of growth factors, changes in pH, hypoxia, and angiogenesis. Ca^2+^ can also modulate tumor invasion and migration and it is crucial in cell cycle stages (12). Therefore, imbalances in Ca^2+^ homeostasis can trigger important changes in the cellular profile that contribute to development of cells with malignant features (13).

Different types of Ca^2+^ channels, exchangers, and pumps are important to control intracellular Ca^2+^ concentration. Voltage-dependent ion channels are commonly associated to excitable cells, but the expression of T-type voltage-dependent Ca^2+^ channels (Ca_V_3) has been reported in various types of non-excitable cells, including breast cells. Ca_V_3 are membrane proteins formed by one or more subunits, including the pore-forming α1 subunit, that can be encoded by different genes (CACNA1G; CACNA1H; CACNA1I), resulting in three different protein isoforms, Ca_V_3.1, Ca_V_3.2, and Ca_V_3.3, respectively (14,15).

Ca_V_3 mRNA expression in breast cancer is still poorly understood. An increased expression of Ca_V_3.1 and Ca_V_3.3 (16) has been reported, but decreased expression of Ca_V_3.1 and Ca_V_3.2 (17) has also been reported in invasive mammary carcinoma tissues. One possible reason for these inconsistencies may be related to the histological types of breast cancer, which have different characteristics of development and progression (16,17).

There is evidence that blockage of Ca_V_3 channels in MCF-7 cells induces cell death (18). Ca_V_3.1 and Ca_V_3.2 silencing can lead to decreased proliferation rate (19), demonstrating a possible role in cell proliferation control. Corroborating this idea, a study carried out on MCF-7 cells using extracellular pressure to simulate the role exerted by the stroma in tumors showed that Ca^2+^ influx mediated by Ca_V_3.3 recruited pathways that stimulated cell proliferation (20). On the other hand, silencing and/or blocking of Ca_V_3.1 in MCF-7 cells increased proliferation and decreased the number of cells in apoptosis, suggesting a possible function as a tumor suppressor gene (21).

The differences between previous works indicate that Ca_V_3 may exert a role in tumorigenesis and that its signaling pathways still need to be further investigated. Furthermore, the behavior of Ca_V_3 protein in the breast epithelium without alteration (BWA) and in proliferative non-neoplastic and neoplastic ductal epithelial lesions of the human breast has not been investigated. These analyses can provide preliminary information on the role of Ca_V_3 in tumor transformation of breast cancer from pre-neoplastic and neoplastic lesions. Therefore, this study aimed to analyze protein expression and subcellular localization of Ca_V_3.1, Ca_V_3.2, and Ca_V_3.3 in tissues from BWA, ductal hyperplasia (DH), DCIS, and IDC.

## Material and Methods

### Ethical issues

This research was conducted following the ethical principles for the use of human material as a primary source of information after approval from the Research Ethics Committees (Comitê de Ética em Pesquisa - COEP) of Federal University of Minas Gerais and of Hospital Santa Casa de Misericórdia de Belo Horizonte (33481919.4.0000.5149 and 33481919.4.3001.5138, respectively).

### Histopathological evaluation

Fifty-six samples of human breast stored in paraffin blocks at the Hospital Santa Casa de Misericordia de Belo Horizonte, collected between 2010 and 2015, were analyzed. Histological samples of human breast stained with hematoxylin-eosin were examined under optical microscopy for classification into BWA, DH, DISC, or IDC, according to the guidelines of the World Health Organization. Regions were classified and analyzed individually, because samples from one individual may contain several lesions in addition to the normal mammary epithelium.

### Immunohistochemistry

Immunohistochemistry was performed using the peroxidase reaction technique with identification by polymerized secondary antibody (Novocastra Post Primary and Novolink Polymer; Leica Biosystems, UK). The antigen retrieval was performed by pressurized humid heat at 137°C (Autoclave ALT 5LD Plus; ALT, Brazil) with Target Retrieval Solution Citrate - low pH (Agilent Technologies, USA). Blockage of endogenous peroxidase and endogenous proteins was done with Novocastra reagents (Leica Biosystems). Antibodies anti-Ca_V_3.1, -Ca_V_3.2, and -Ca_V_3.3 (ACC-021, ACC-025, and ACC-009; Polyclonal, Alomone Labs, Israel) were used in dilutions of 1:250, 1:100 and 1:200, respectively. Incubation with primary antibody was set at 16 h and with 3'3-diaminobenzidine chromogen Novocastra DAB Chromogen (Leica Biosystems) at 1 min. Sections were counterstained with hematoxylin dye. For positive controls of Ca_V_ 3.1 and Ca_V_3.2 labelling, we used samples of human skeletal muscle, and for Ca_V_3.3, samples of human brain were used. For negative controls, the primary antibody incubation step was replaced by incubation with immunoglobulins of the same species as the primary antibody.

### Interpretation of immunohistochemistry findings

For statistical analyses, previously classified regions were categorized into scores, as follows: BWA: 1; DH: 2; DCIS: 3; IDC: 4. All histological analyses were performed using conventional optical microscopy (Olympus BX41, Japan), with 40× objective, and the images were captured from a system using a SPOT 3.4.5 Basic^®^ camera (Spot Insight QE Microscope Camera 2 Mp; Spot Imaging, USA) adapted to the optical microscopy. Some samples were excluded for certain markers because they did not show immunohistochemical reactivity.

Membrane, cytoplasmic, and nuclear immunohistochemical staining was analyzed for each of the antibodies used. Membrane expression was classified by the percentage of cells that had immunoreaction, following the scoring: 0 (absence), 1+ (<25%), 2+ (25-50%), and 3+ (>50%). Cytoplasmic staining was analyzed according to expression intensity, using the scoring: 0 (absence), 1+ (weak), 2+ (moderate), and 3+ (strong). For nuclear expression analysis, the percentage of stained cells with weak, moderate, or strong expression intensity was determined. Then, data was separated into two groups: negative expression, encompassing the cells with no expression and/or weak intensity; and positive expression, encompassing the cells with moderate and strong intensity. Also, the predominant percentage of cells with nuclear staining was determined, considering: 0 (absence), 1+ (weak), 2+ (moderate), and 3+ (strong).

### Statistical analysis

Statistical analysis was performed with GraphPad Prism v. 8.0 (GraphPad Software, USA). The normality of data was tested by the Kolmogorov-Smirnov test. Possible correlations were assessed using Pearson's correlation coefficient. Results were considered significant when P<0.05.

## Results

**Figure 1:**
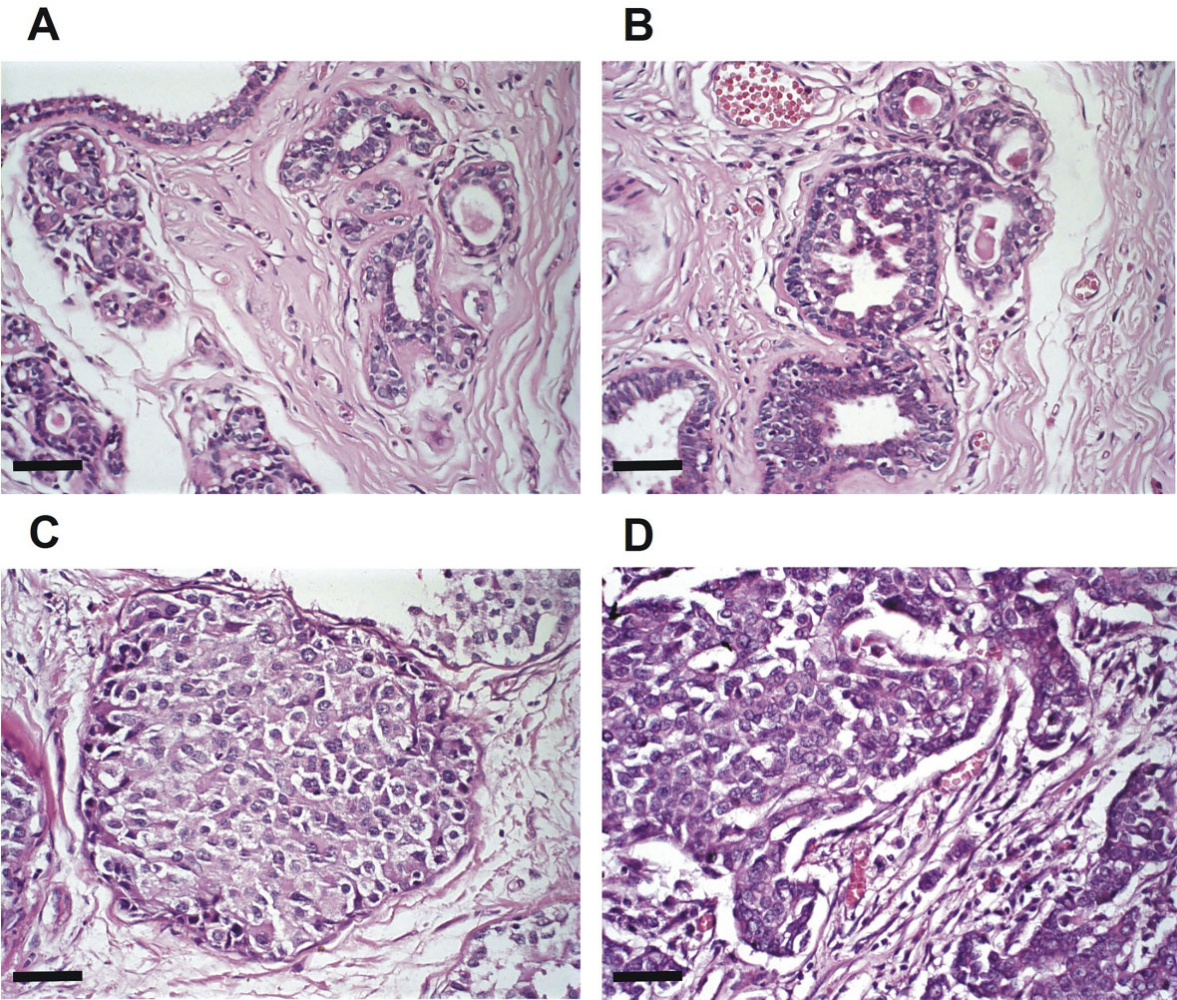
Classification of histological types. Mammary glands, stained with hematoxylin and eosin (400× magnification, scale bar, 50 µm). **A**, Breast without alteration; (**B**) ductal hyperplasia; (**C**) ductal carcinoma in situ; (**D**) invasive ductal carcinoma.

Histopathological analyses were conducted in 56 different cases, where different regions were analyzed and classified regarding the lesion types found in each sample. Eleven regions of BWA were analyzed, being considered only those with two or more mammary lobes without histological alterations (Figure 1A). DH were considered when at least two ductal regions presented three or more layers of epithelial cells above the basal membrane with or without intraductal projections, with no distinction of nuclear alterations, and with a predominance of nuclear monomorphism (Figure 1B), accounting for a total of 18 regions. Fifteen DCIS regions were selected, considering the presence of epithelial cell proliferation with discrete to moderate nuclear pleomorphism and evident nucleoli and the formation of intraductal projections or solid masses restricted to the terminal ductal lobular units, surrounded by a myoepithelial cell layer (Figure 1C). IDC was identified in 24 regions, being evaluated areas with atypical epithelial proliferation, creating isolated cell nests in mammary stroma, with the presence of moderate to high nuclear pleomorphism and a high number of mitosis (Figure 1D).

**Figure 2:**
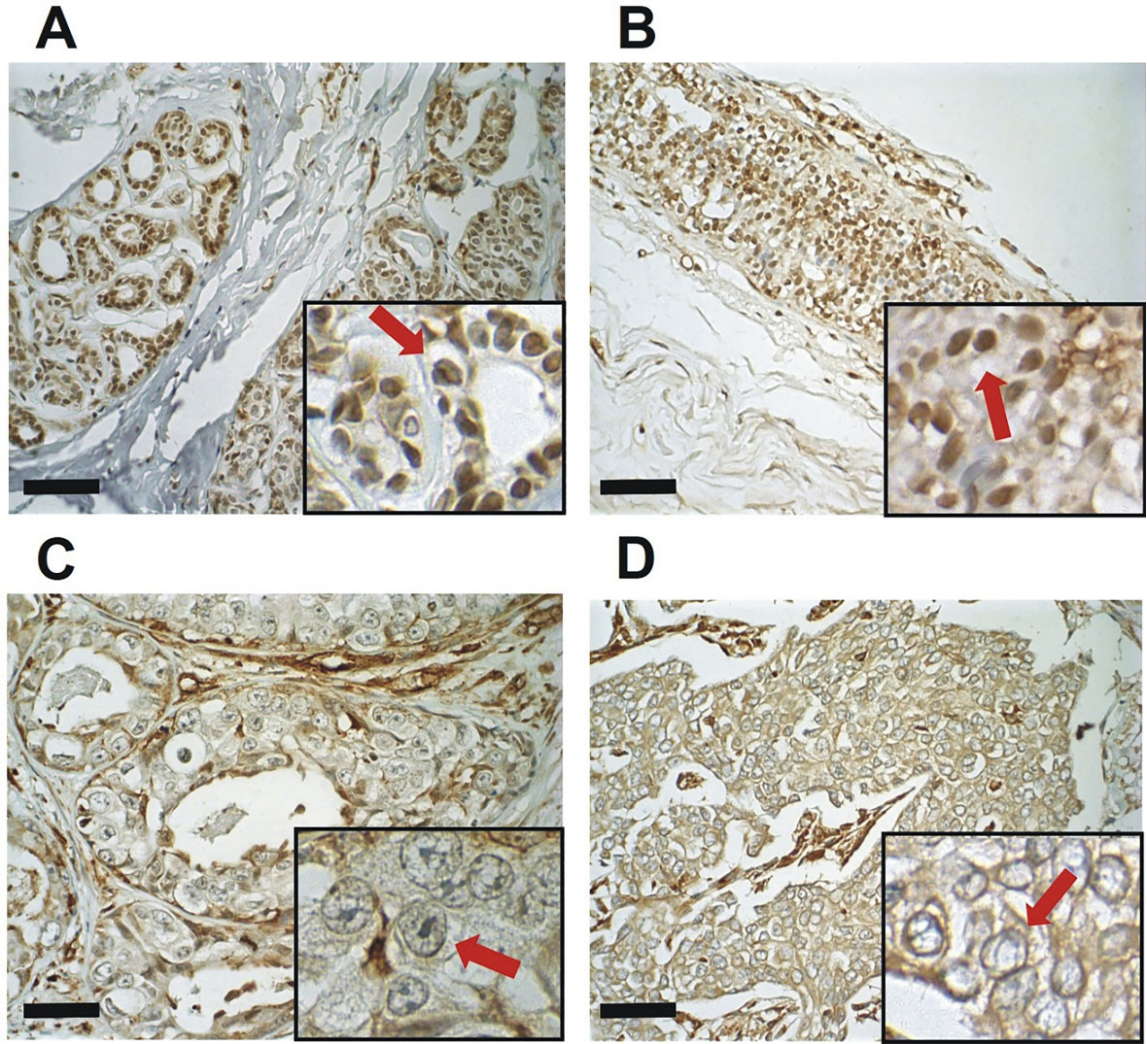
Human mammary glands with immunohistochemical staining for Ca_V_3.1 identified by DAB chromogen staining and hematoxylin counterstaining (main image 400× magnification, insert 600× magnification; scale bar, 50 µm). **A**, Breast without alteration showing nuclear expression (arrow) and absence of cytoplasmic immunostaining. **B**, Ductal hyperplasia with nuclear immunostaining (arrow). **C**, Ductal carcinoma *in situ* showing decreased nuclear expression and presence of cytoplasmic expression (arrow). **D**, Invasive ductal carcinoma with few nuclear immunostainings and cytoplasmic expression (arrow). Statistical test: Pearson’s correlation coefficient.

Ca_V_3.1 immunohistochemical staining (Figure 2) indicated a negative correlation between predominant nuclear expression and histological classification score (r=-0.73; P<0.05). Regarding cytoplasmic expression analysis, most cases of BWA (87.5%; 7/8) and DH (80.0%; 8/10) did not present this expression, except for one BWA case (12.5%; 1/8) with weak expression and two DH cases (20.0%; 2/10) with moderate cytoplasmic expression. However, DCIS presented a different pattern with a greater number of cases with weak cytoplasmic intensity (60.0%; 6/10) and only one (10.0%; 1/10) with moderate. Similar to this, IDC presented cases between weak (31.5%; 6/19) and moderate (42.1%; 8/19) expression (Table 1). In line with these data, a positive correlation was found between cytoplasmic expression and histological score (r=0.49; P<0.05) and a negative correlation between predominant nuclear expression and cytoplasmic expression (r=-0.42; P<0.05).

**Table 1:** Percentage (number) of cases with cytoplasmic immunostaining categorized as absent expression, and weak, moderate, or strong expression of Ca_V_3.1.

	Absent	Weak	Moderate	Strong
Mammary gland without alteration	87.5% (7/8)	12.5% (1/8)	-	-
Ductal hyperplasia	80.0% (8/10)	-	20.0% (2/10)	-
Ductal carcinoma *in situ*	30.0% (3/10)	60.0% (6/10)	10.0% (1/10)	-
Invasive ductal carcinoma	26.3% (5/19)	31.5% (6/19)	42.1% (8/19)	-

**Figure 3:**
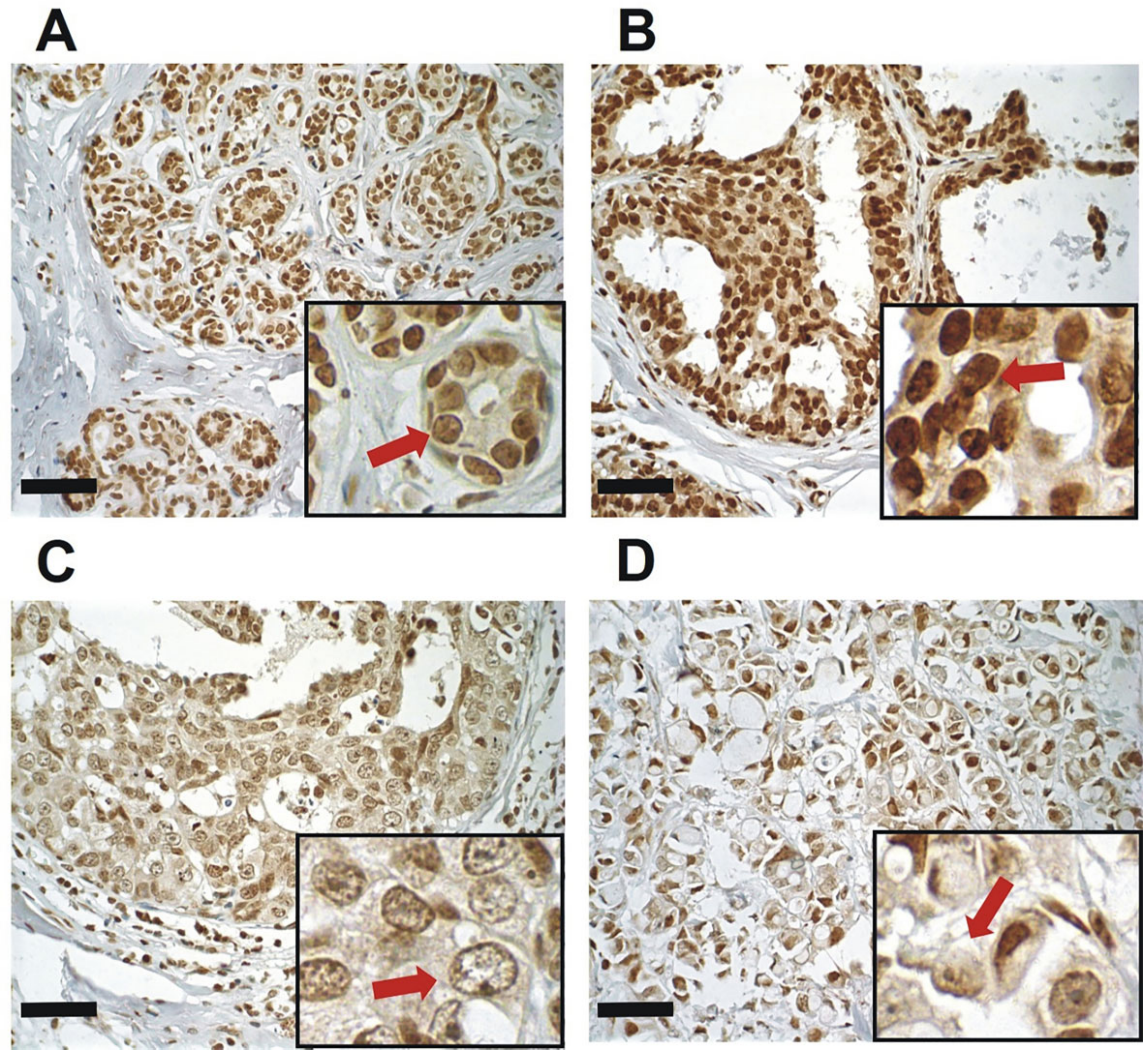
Human mammary glands with immunohistochemical staining for Ca_V_3.2 identified by DAB chromogen staining and hematoxylin counterstaining (main image 400× magnification and insert 600× magnification; scale bar, 50 µm). **A**, Breast without alteration showing nuclear expression (arrow) and no cytoplasmic immunostaining. **B**, Ductal hyperplasia with nuclear expression (arrow). **C**, Ductal carcinoma *in situ* showing decreased nuclear expression and cytoplasmic immunostaining (arrow). **D**, Invasive ductal carcinoma with few nuclear immunostainings and cytoplasmic expression (arrow). Statistical test: Pearson’s correlation coefficient.

In Ca_V_3.2 immunohistochemical analysis (Figure 3), a negative correlation was observed between predominant nuclear expression and histological score (r=-0.41; P<0.05). All histological classifications analyzed had Ca_V_3.2 cytoplasmic expression. All cases (DH: 75.0%, 12/16; DISC: 76.9%, 10/13; IDC: 59.0%, 13/22) had predominance of weak expression, thus the emphasis was on BWA, where all regions were classified as having weak expression (100.0%; 9/9). For DH, two cases had no expression (12.5%; 2/16) and two, moderate expression (12.5%; 2/16). In DCIS, moderate staining was also seen (23.0%; 3/13). In IDC, the number of regions with weak and moderate expression (59.0%; 13/22 and 36.3%; 8/22, respectively) was better distributed compared to other types of lesions, also having a case with no expression (4.54%, 1/22) (Table 2). Corroborating these data, a positive correlation was seen between Ca_V_3.2 cytoplasmic expression and histological score (r=0.28; P<0.05).

**Table 2:** Percentage (number) of cases with cytoplasmic immunostaining categorized as absent expression, and weak, moderate, or strong expression of Ca_V_3.2.

	Absent	Weak	Moderate	Strong
Mammary gland without alteration	-	100.0% (9/9)	-	-
Ductal hyperplasia	12.5% (2/16)	75.0% (12/16)	12.5% (2/16)	-
Ductal carcinoma *in situ*	-	76.9% (10/13)	23.0% (3/13)	-
Invasive ductal carcinoma	4.5% (1/22)	59.0% (13/22)	36.3% (8/22)	-

**Figure 4:**
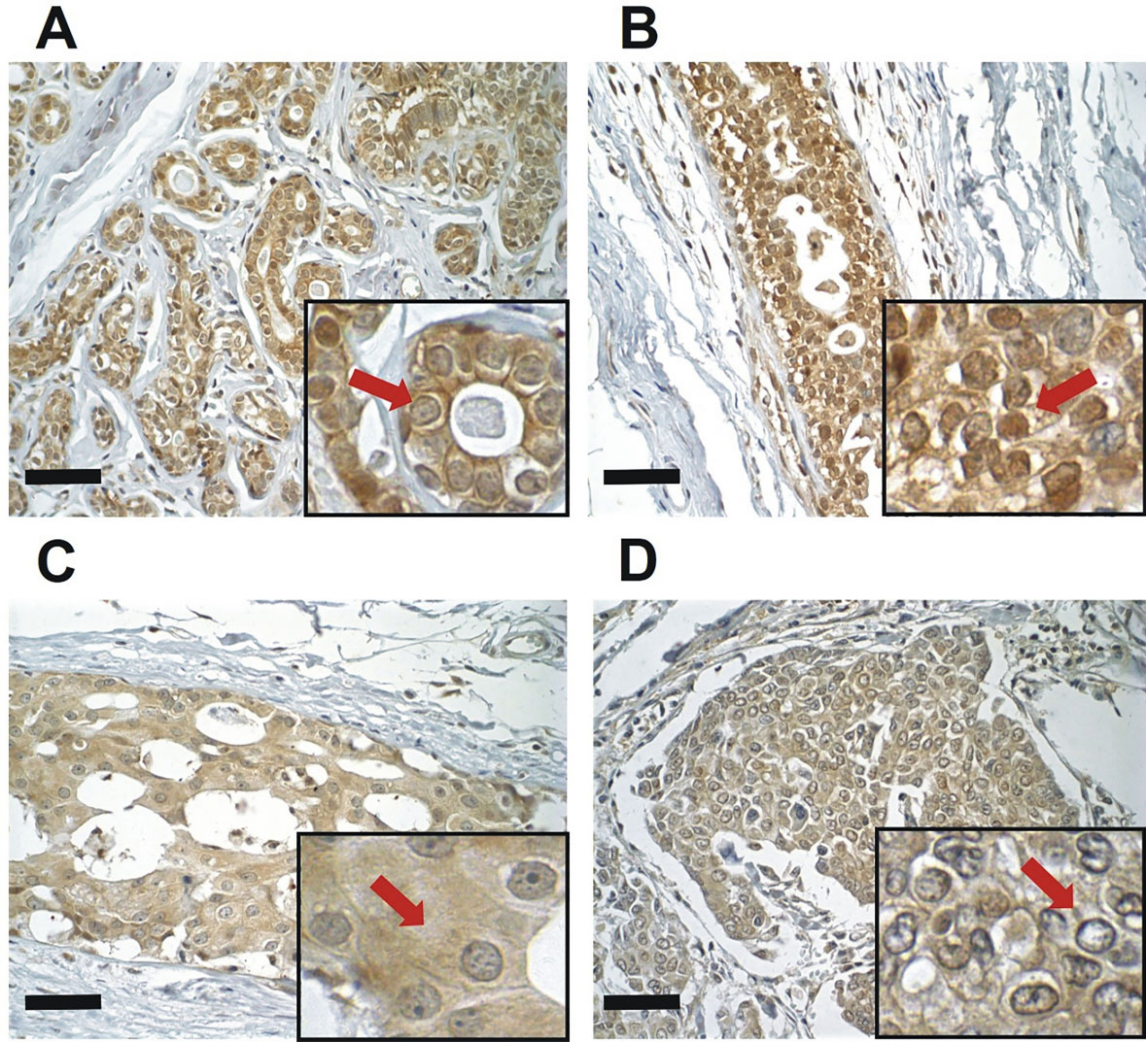
Human mammary glands with immunohistochemical staining for Ca_V_3.3 identified by DAB chromogen staining and hematoxylin counterstaining (main image 400× and insert 600× magnification; scale bar, 50 µm). **A**, Breast without alteration showing nuclear expression (arrow) and no cytoplasmic immunostaining. **B**, Ductal hyperplasia with nuclear expression (arrow). **C**, Ductal carcinoma *in situ* showing decreased nuclear expression and cytoplasmic expression (arrow). **D**, Invasive ductal carcinoma with few nuclear immunostainings and cytoplasmic expression (arrow). Statistical test: Pearson’s correlation coefficient.

Analysis of Ca_V_3.3 immunostaining (Figure 4) also presented a negative correlation between predominant nuclear expression and histological score (r=-0.49; P<0.05). Ca_V_3.3 cytoplasmic expression was also observed in all histological classifications. BWA cases presented moderate (66.6%; 6/9) or weak staining (33.3%; 3/9). DH also showed weak and moderate expressions (46.6%; 7/15, for each), but one case had a strong expression (6.6%; 1/15). DCIS had a greater number of cases with weak expression (70.0%; 7/10) and a smaller number with moderate (30.0%; 3/10), while IDC had a similar number of cases with weak (52.3%; 11/21) and moderate (42.8%; 9/21) expressions, and only one case with no expression (4.7%; 1/21) (Table 3). The Ca_V_3.3 was the only one of the Ca_V_ proteins with a plasma membrane expression. This expression was found in three cases of BWA, with one of them (11.1%; 1/9) presenting immunostaining in up to 25% of the cells and the other two (22.2%; 2/9) presenting positivity in 25 to 50% of cells. In addition to these, only one case of DH (6.6%; 1/15) had 25 to 50% of positive cells. The membrane expression was negatively correlated with histological score (r=-0.34; P<0.05).

**Table 3:** Percentage (number) of cases with cytoplasmic immunostaining categorized as absent expression, and weak, moderate, or strong intensity of expression for Ca_V_3.3.

	Absent	Weak	Moderate	Strong
Mammary gland without alteration	-	33.3% (3/9)	66.6% (6/9)	-
Ductal hyperplasia	-	46.6% (7/15)	46.6% (7/15)	6.6% (1/15)
Ductal carcinoma *in situ*	-	70.0% (7/10)	30.0% (3/10)	-
Invasive ductal carcinoma	4.7% (1/21)	50.3% (11/21)	42.8% (9/21)	-

## Discussion

In the present study, the expression pattern and subcellular localization of Ca_V_3 isoforms varied among neoplastic lesions, non-neoplastic ductal proliferative epithelial lesions, and normal mammary epithelium. Ca_V_3 channels had higher nuclear immunostaining in normal and hyperplastic mammary epithelium than in neoplastic epithelial lesions. Thus, cytoplasmic expression of Ca_V_3.1 and Ca_V_3.2 seemed to follow a pattern as mammary epithelium transforms into pre-malignant and malignant stages. Our results suggested that alterations in subcellular localization of Ca_V_3 can be associated with different roles in tumorigenesis.

Changes in the expression of other Ca^2+^ channels have been found in breast cancer (12). The different subcellular localization presented by our research were also observed at TRPM8 in human prostate cancer cells. Although these channels were expressed in the endoplasmic reticulum (ER) membrane and not in the cellular membrane, they were overexpressed in TRPM8 HEK-293 cells in both plasma and ER membranes, and these changes seem to contribute to tumorigenesis through changes in Ca^2+^ signaling (22,23). In addition, it has been reported that an increase in ORAI1 expression promotes intracellular Ca^2+^ oscillations leading to activation of proliferative signaling pathways in esophageal squamous carcinoma cells (24).

Increased Ca_V_3.2 mRNA has been observed in trastuzumab-resistant breast cancer cells, however it does not seem to be a driver in this resistance (25). However, Ca_V_3 knockdown was associated with drug-resistant cancer therapy (26). Ca^2+^ influx through the cellular membrane is the most well-known function of Ca_V_3 (27). Unexpectedly, we did not detect Ca_V_3 in the plasma membrane. We then reasoned that Ca^2+^ influx through these channels may not be a necessary condition to neoplastic transformation in breast cancer. The concept that ion channels have non-canonical functions was appreciated only recently and provides evidence that these proteins can act in other cellular processes, such as proliferation control, through their interaction with cyclins and other cell cycle proteins (28,29).

It is reasonable to think that the cytoplasmic immunostaining observed for these channels may be related with cellular trafficking of these proteins between different subcellular compartments, like ER, which results in their no longer being expressed in the cell membrane (30). Trafficking can be influenced by mutations in channel protein structure or by interaction with other proteins, such as adaptor proteins from Stac family and Kelch-like 1, an actin-organizing protein (31,32). Ion channel expression in cellular organelles has already been described. At the mitochondria inner membrane, the presence of K^+^ channels was associated to apoptosis inhibition (33), while in lysosomes, the influx of Ca^2+^ ions can modulate autophagy, an important process for tumor cell survival (34). BCL-2 overexpression in MCF-7 cells seems to modulate IP3 receptors in ER, leading to a leakage of luminal Ca^2+^ and consequently inhibiting the intrinsic apoptosis pathway (22,35). To our knowledge, no studies have reported the presence of Ca_V_3 in subcellular compartments, but this apoptosis theory would be a possible explanation for our findings.

In the nucleus, Ca^2+^ channels can act as a second messenger through Ca^2+^ ions. After Ca^2+^ influx, transcription factors NFAT and NFκB can be translocated to the nucleus and act in the transcription of genes related to cell survival, proliferation, and immune response (36). Also, Ca_V_3 inhibition may result in cell cycle arrest in G1 or G2 (37). Otherwise, some Ca^2+^ channels such as ryanodine receptors and IP3 receptors, which are types of ligand-gated Ca^2+^ channels, can be found in the nucleus, but their function in nuclear Ca^2+^ signaling is not yet elucidated (36). In addition, gene transcription regulation of Ca_V_1.2 channels has been demonstrated. This effect is related to the proteolytic cleavage of the 74 kDa peptide from the C-terminal of the α1-subunit that translocates to the nucleus to act as a transcriptional regulator (38). Despite this, the role of Ca_V_3 in the cell nucleus is not yet known.

Our results suggested that Ca_V_3 channels are involved, in an unknown way, in the progression of non-neoplastic proliferative epithelial lesions of the breast into neoplastic lesions, composed of cells with loss of differentiation. Such involvement in cell differentiation has already been seen in mouse pre-adipocytes, where increased expression of Ca_V_3.1 was observed compared with differentiated adipocytes (39). This relationship has also been seen in prostate cancer, in which tumors progress with neuroendocrine differentiation, a more aggressive tumor, with higher proliferation rates and worse prognosis. Studies demonstrated that there was an increase in Ca_V_3.2 expression in cell lines with neuroendocrine differentiation compared to prostate cancer, indicating that this channel has a role in morphological and biochemical changes of prostate tumor cells (40).

In conclusion, our results suggested that as the mammary epithelium advanced in the neoplastic transformation model for invasive ductal carcinoma a decrease in nuclear expression of Ca_V_3 channels was observed. In parallel, there was no increase in Ca_V_3.1 and Ca_V_3.2 cytoplasmic expression. These promising findings should encourage further studies to elucidate the role of these channels in neoplastic transformation of breast tumor. Therefore, understanding the localization, expression, and dynamics of these channels will contribute to both early detection of non-neoplastic lesions and to investigation of novel potential therapeutic targets in breast cancer.
